# Formononetin inhibits IgE by huPlasma/PBMCs and mast cells/basophil activation via JAK/STAT/PI3-Akt pathways

**DOI:** 10.3389/fimmu.2024.1427563

**Published:** 2024-08-15

**Authors:** Ibrahim Musa, Zhen-Zhen Wang, Nan Yang, Xiu-Min Li

**Affiliations:** ^1^ Department of Pathology Microbiology & Immunology, New York Medical College, New York, NY, United States; ^2^ Academy of Chinese Medical Science, Henan University of Chinese Medicine, Zhengzhou, China; ^3^ R&D Division, General Nutraceutical Technology LLC, Elmsford, NY, United States; ^4^ Department of Otolaryngology, School of Medicine, New York Medical College, New York, NY, United States

**Keywords:** formononetin, food anaphylaxis, computational modeling, target mining, molecular docking, gene ontology enrichment

## Abstract

**Rationale:**

Food allergy is a prevalent disease in the U.S., affecting nearly 30 million people. The primary management strategy for this condition is food avoidance, as limited treatment options are available. The elevation of pathologic IgE and over-reactive mast cells/basophils is a central factor in food allergy anaphylaxis. This study aims to comprehensively evaluate the potential therapeutic mechanisms of a small molecule compound called formononetin in regulating IgE and mast cell activation.

**Methods:**

In this study, we determined the inhibitory effect of formononetin on the production of human IgE from peripheral blood mononuclear cells of food-allergic patients using ELISA. We also measured formononetin’s effect on preventing mast cell degranulation in RBL-2H3 and KU812 cells using beta-hexosaminidase assay. To identify potential targets of formononetin in IgE-mediated diseases, mast cell disorders, and food allergies, we utilized computational modeling to analyze mechanistic targets of formononetin from various databases, including SEA, Swiss Target Prediction, PubChem, Gene Cards, and Mala Cards. We generated a KEGG pathway, Gene Ontology, and Compound Target Pathway Disease Network using these targets. Finally, we used qRT-PCR to measure the gene expression of selected targets in KU812 and U266 cell lines.

**Results:**

Formononetin significantly decreased IgE production in IgE-producing human myeloma cells and PBMCs from food-allergic patients in a dose-dependent manner without cytotoxicity. Formononetin decreased beta-hexosaminidase release in RBL-2H3 cells and KU812 cells. Formononetin regulates 25 targets in food allergy, 51 in IgE diseases, and 19 in mast cell diseases. KEGG pathway and gene ontology analysis of targets showed that formononetin regulated disease pathways, primary immunodeficiency, Epstein-Barr Virus, and pathways in cancer. The biological processes regulated by formononetin include B cell proliferation, differentiation, immune response, and activation processes. Compound target pathway disease network identified NFKB1, NFKBIA, STAT1, STAT3, CCND1, TP53, TYK2, and CASP8 as the top targets regulated at a high degree by formononetin. TP53, STAT3, PTPRC, IL2, and CD19 were identified as the proteins mostly targeted by formononetin. qPCR validated genes of Formononetin molecular targets of IgE regulation in U266 cells and KU812 cells. In U266 cells, formononetin was found to significantly increase the gene expression of NFKBIA, TP53, and BCL-2 while decreasing the gene expression of BTK TYK, CASP8, STAT3, CCND1, STAT1, NFKB1, IL7R. In basophils KU812 cells, formononetin significantly increased the gene expression of NFKBIA, TP53, and BCL-2 while decreasing the gene expression of BTK, TYK, CASP8, STAT3, CCND1, STAT1, NFKB1, IL7R.

**Conclusion:**

These findings comprehensively present formononetin’s mechanisms in regulating IgE production in plasma cells and degranulation in mast cells.

## 1 Introduction

Food allergy or anaphylaxis is an immune disorder that can be separated into two types: Immunoglobulin E (IgE) mediated and non-IgE mediated allergic disorders (1). IgE is the least abundant immunoglobulin isotype in the serum, present at a concentration level of 0.1 to >100ku/L. However, in people with food allergy, allergen-specific IgE levels have been shown to be increased by up to ten times the normal levels (2). In an individual with food allergy, antigen-presenting cells uptake the allergenic protein, break it down into peptides, present it to T cells, and activate them to produce two key signals, cytokines IL-4 and IL-13. These cytokines activate class switch recombination in naïve B cells, turning them into IgE-producing plasma B cells. The allergen-specific IgE produced then binds to high-affinity IgE receptors known as FcϵRI on mast cells, basophils, epidermal and Langerhans cells (3). Upon re-exposure to the same allergenic protein, the allergen-specific IgE bound to FcϵRI on mast cells will bind to the allergenic protein, triggering mast cell degranulation and the subsequent release of mediators such as histamine, tryptase, leukotrienes, and prostaglandins, causing symptoms of food anaphylaxis, such as nausea, vomiting, constriction of airways, hives, itching, and low blood pressure (3).

Currently, there are limited therapies available for the prevention and treatment of food anaphylaxis. Current treatments, such as epinephrine and corticosteroids, are for acute management and do not prevent or treat immune disorders. Long-term treatments are being developed, including recently approved non-allergen-specific immunotherapy options such as Omalizumab, Dupilumab, and Ekotimab. However, there is a lack of relevant results regarding long-term sustained unresponsiveness. Other non-allergen-specific immunotherapies, such as probiotics and symbiotics, require more studies and relevant results. Allergen-specific immunotherapy Palforzia, an FDA-approved oral immunotherapy, shows promising results. However, it is time-consuming and can result in serious side effects. Therefore, there is an ongoing search for better treatment options. The most promising candidates for the treatment and prevention of food allergy should be therapeutics that prevent IgE production in plasma B cells and prevent mast cell degranulation.

Formononetin is an O-methylated isoflavone present in herbs such as *Sophorae flavescentis* (Ku-Shen) and species of clovers, such as red clover (4). Studies have shown that formononetin has multiple therapeutic effects, including anticancer, anti-inflammatory, antimicrobial, and antihypertensive effects. Flavonoids are an important class of natural products with favorable biochemical, antioxidant, anti-inflammatory, anti-mutagenic, and anti-carcinogenic effects. Recent studies have also shown that they can decrease IgE. Chrysin and apigenin are examples of flavonoids that have been shown to decrease serum IgE (5). Other flavonoids, such as quercetin, myricetin, and luteolin, have been shown to decrease serum IgE antibodies in mice sensitized to OVA (6–8). Therefore, we conducted studies to identify the potential mechanism of formononetin in preventing food anaphylaxis due to previous studies showing its IgE inhibitory effect and other studies showing that other flavonoids have IgE inhibitory effects (9). There are still gaps in knowledge on the mechanisms of IgE inhibition and mast cell degranulation by potential therapeutics and this study aims to bridge it.

**Figure 1:**
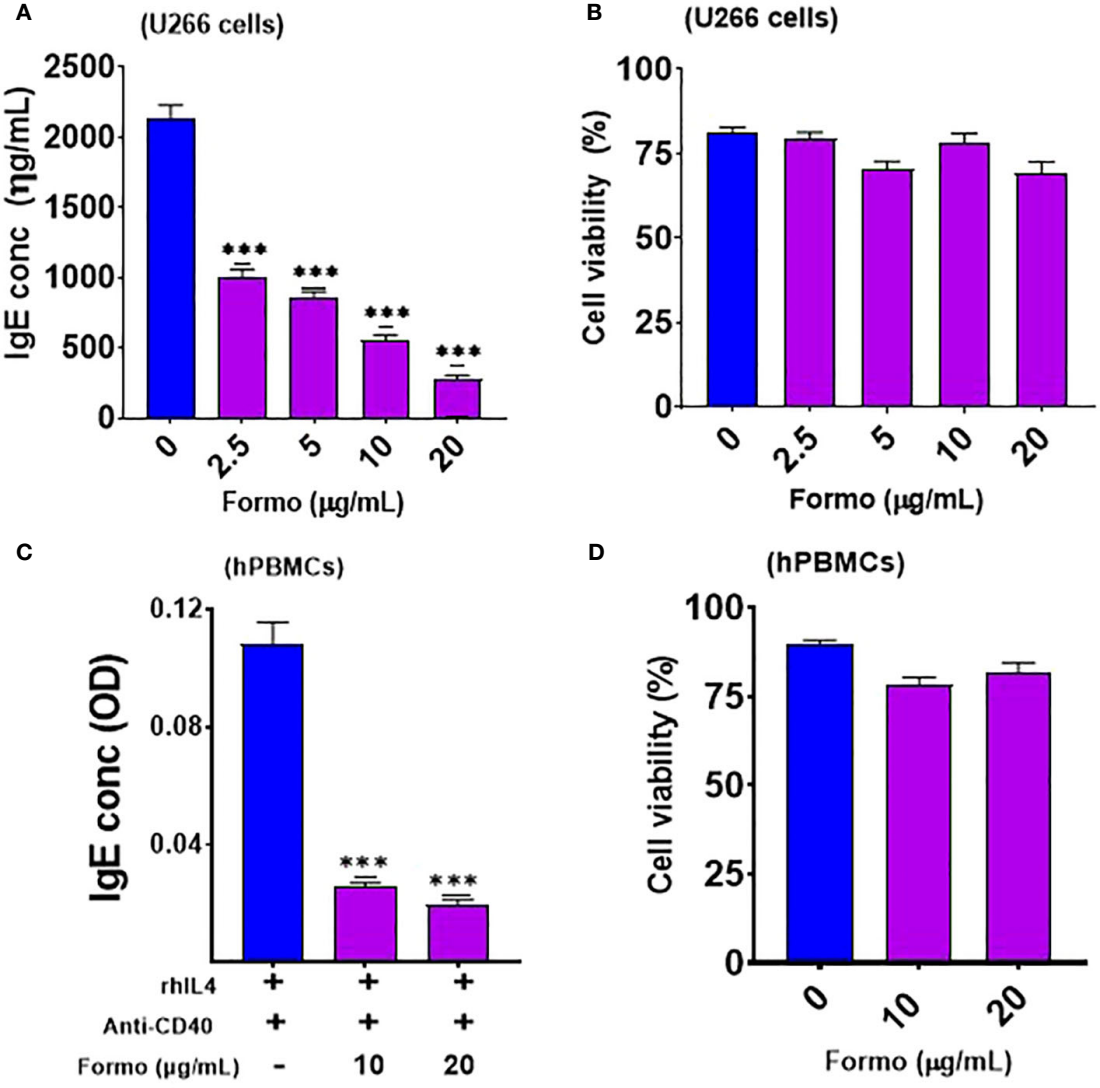
Formononetin inhibits IgE production in IgE producing Human Myeloma cells and Peripheral Blood Mononuclear cells in a dose dependent manner without cytotoxicity. **(A)** IgE inhibitory effect of formononetin cultured across a concentration of 20, 10, 5, 2.5 µg/ml with IgE producing human myeloma cells for 72 hours **(B)**. Cell viability of IgE producing human myeloma cells cultured with formononetin across different concentrations for 72 hours determined by Trypan Blue Assay. **(C)** IgE inhibitory effect of formononetin cultured across a concentration of 20, 10 µg/mL with peripheral blood mononuclear cells collected from food allergic patients for 72 hours **(D)**. Cell viability of peripheral blood mononuclear cells cultured with formononetin across different concentrations for 72 hours determined by Trypan Blue Assay. Data represents triplicate experiments and expressed as mean ±SD. ***p<0.001 vs control (0.1%DMSO). U266; IgE producing human myeloma cell line; hPBMCs; human peripheral blood mononuclear cells. Formo, Formononetin.

Drug discovery has been aided by newer technologies, specifically those that identify potential targets of novel treatments in different diseases. Computational technology is used to identify potential targets of novel molecules in diseases, and with these targets, they can construct the potential mechanism of action of the new treatment. This helps to accelerate the preclinical *in vitro* and *in vivo* studies to determine if the potential treatment can proceed to clinical trials. In this study, we employed the use of computational technology to identify potential therapeutic targets of formononetin, which has been previously shown to inhibit IgE production (10). We used target platforms such as Malacards, GeneCard, and SEA, Swiss Target to obtain biological and disease targets, respectively. From the obtained targets, we constructed a gene ontology, KEGG pathway, protein-protein interaction network. **Figure 1** shows the flow chart of this study. The targets obtained from the computational modeling were validated *in vitro* using the IgE producing multiple myeloma cells known as U266 cells and basophilic leukemia cell line, KU812.

## 2 Materials and methods

### 2.1 Human IgE-producing myeloma cell culture and formononetin *in vitro* treatment

Plasma cell line, U266 cells were used to evaluate the IgE inhibitory effect of formononetin (Gift provided by General Nutraceutical Technology, Elmsford, NY). The chemical structure and HPLC analysis of the quality are shown in **Supplementary Figure 1** (**Supplementary Figure 1**). U266 cells are B lymphocytes isolated from the peripheral blood of a male patient (53 years old) with myeloma cancer. They are widely used in immunology research to investigate the IgE inhibitory effect of new compounds, herbs, and extracts. We purchased the U266 cells from American Type Culture Collection in Rockville, Maryland. To culture the cells, we used RPMI 1640 media with 10% Fetal Bovine Serum, 1 mmol/L of Sodium, 0.5% penicillin-streptomycin, and 1x10^-5^ mol/L of β-mercaptoethanol from Gibco (ThermoFisher Scientific, Waltham, MA). Once we achieved the desired cell concentration of 1.0 x 10^6^ cells/mL, we cultured the U266 cells at with formononetin over a range of concentrations (20, 10, 5, and 2.5 µg/mL) for the treated group. We used 0.1% DMSO for the untreated group. The culture was incubated at 37°C and 5% CO_2_ for 72 hours. After the culture period, we harvested the supernatant and measured Immunoglobulin E levels using ELISA. We also determined cell viability using the trypan blue exclusion assay (11).

### 2.2 Isolation of peripheral blood mononuclear cells from food allergic patients and cell culture, and formononetin *in vitro* treatment

Blood samples were obtained from patients between the ages of 10 to 13 years old, who had been diagnosed with food allergies by a physician. The samples included both male and female patients. These patients had previously experienced allergic reactions to food allergens like peanuts and tree nuts. The Institutional Review Board of New York Medical College approved this study, and all patients provided informed consent before participation. Ficoll Hypaque from Pharmacia, Piscataway, NJ was used to isolate peripheral blood mononuclear cells (PBMCs) from the collected blood samples. The blood samples were spun at 1000 rpm for 15 minutes at 20˚C to separate the plasma. The top plasma layer was aspirated, and phosphate-buffered saline (PBS) 1X was added to the tube. PBMCs were isolated using Ficoll Hypaque and centrifugation. The isolated PBMCs were then suspended in RPMI 1640, Gibco from ThermoFisher Scientific, Waltham, MA, with 10% Fetal Bovine Serum, Gibco TM, 1 mmol/L of Sodium, Gibco, 0.5% penicillin-streptomycin, Gibco, and 1x10^-5^ mol/L of β-mercaptoethanol, Gibco.1.0 x 10^6^ cells/mL of PBMCs were stimulated with 100ng/mL human recombinant interleukin 4 (R&D systems, Minneapolis, Minnesota), 1 µg/mL anti-CD40 monoclonal antibody (R&D systems, Minneapolis, Minnesota), and cultured in the presence or absence of formononetin at 10 and 20 µg/mL for 10 days (12). After the culture period, the supernatant was collected, and IgE levels were determined using ELISA. Cell viability was determined via the trypan blue assay.

### 2.3 β-Hexosaminidase enzymatic release

RBL-2H3 cells were plated at a density of 2.5 x 10^5^ cells per well in 24-well plates. Formononetin was added to the cells at concentrations of 10 and 20 μg/mL, and the cells were incubated for 24 or 48 hours. The cells were then sensitized with 72 ng/mL of anti-DNP IgE and challenged with 150 μg/mL of DNP-BSA. Degranulation was measured by determining β-hexosaminidase activity, following published methods (13). The following experiment involved plating KU812 cells in 24-well plates at a density of 1.0 x 106 cells per well. The cells were incubated with Formononetin at concentrations of 10 and 20 μg/mL for 24 hours. Afterwards, the cells were sensitized using serum containing peanut-specific immunoglobulin E from patients with peanut food allergy and then challenged with crude peanut extract at a concentration of 500 μg/mL. The degranulation was measured using β-hexosaminidase activity, following published methods (13).

### 2.4 Target compilation from databases and repositories

Compound and disease databases provide a wealth of information on targets that can be analyzed to identify new treatment options for various diseases. In this study, we utilized these databases to determine the biological targets of the compound formononetin, and its potential use in treatment and prevention of food anaphylaxis and allergy, IgE and mast cell diseases. We obtained formononetin compound targets from various sources, including literature (14, 15) and databases such as Hit Pick Swiss Target Prediction (16, 17), Similarity Ensemble Approach (18, 19), PubChem (20, 21) and Drug Bank (22). From these sources, we were able to obtain the biological targets of formononetin, while the Therapeutic Target Database (23, 24), Gene Association Database (25), Gene Cards (26, 27), Open Targets Platform (28, 29), and Comparative Toxicogenomic Database (30, 31) were used to identify disease targets. From these we mined the formononetin targets into the disease targets obtained. We then mapped the top 200 genes obtained from each of these databases into the UniProt Database (32, 33) for normalization. These targets were then used for various analysis, including KEGG pathway, Gene Ontology, Compound-Target-Pathway-Disease, and Protein-Protein Interaction Network.

### 2.5 Gene ontology, KEGG and protein-protein interaction analysis

Target enrichment analysis, which includes gene ontology (GO), KEGG pathway, and protein-protein interaction (PPI) analysis, provides a detailed understanding of the molecular mechanisms underlying biological functions. To obtain the GO terms and pathways, the potential targets were mapped to the DAVID database (34, 35) and KOBAS 3.0 database respectively (36, 37). We selected the KEGG pathway and GO biological process terms with a false discovery rate (FDR) lower than 0.01. Additionally, the potential targets were mapped to the String database to obtain their interactions, which were then used to construct the PPI network using Cytoscape (version 3.2.1).

### 2.6 Compound-target-pathway-disease network construction and analysis

After obtaining the targets and identifying significant pathways, C-T-P-D biological networks were created using Cytoscape (v3.2.1). The C-T-P-D network containing formononetin and its related targets for food allergy, IgE and mast cell-related diseases, as well as significant principal pathways. This network provides general information at the molecular level on how formononetin regulates targets in food allergy, IgE, and mast cell diseases. To validate the results obtained from the CTPD network, the Cytoscape plugin, Network Analyzer was used (38).

### 2.7 Computational docking of compounds interaction with targeted therapeutic protein

Molecular docking analysis using Auto Dock Vina (39) was conducted to predict how formononetin binds with biological targets including TYK2, BTK, TP53, BCL2, NFKBIA, and CASP8. The crystal structures of these proteins were obtained from RCSB protein data bank (40, 41) while the formononetin structure was obtained from PubChem without further optimization. To visualize the 3D molecular structure, the PyMOL system (42, 43) and Discovery Studio (44) were used.

### 2.8 Quantitative real-time polymerase chain reaction

Quantitative real-time polymerase chain reaction (RT-PCR) was used to determine the relative gene expression of gene targets. In the treatment group, the test compound formononetin (20 µg/mL) was cultured with U266 cells (1 x 10^6^ cells/mL) while the untreated group was cultured with 0.1% DMSO for 72 hours. After 72 hours, the cells were harvested and washed with PBS. Total RNA was isolated using QIAGEN RNeasy kit, according to the manufacturer’s instructions. The RNA concentration was quantified using a NanoDrop™ 2000/2000c Spectrophotometer. Reverse transcription was performed using ImProm-II Reverse Transcriptase (Promega Corporation, Madison, Wisconsin), according to the manufacturer’s instructions, to obtain the cDNA. The RT-PCR amplification was performed using Maxima SYBR Green qPCR Master Mix kit (Fisher Scientific, Pittsburgh, Pennsylvania). Primers for qRT-PCR were ordered from Integrated DNA Technologies (Coralville, IA), the sequences of the primers are listed in **Supplementary Table 1**.

### 2.9 Western blotting

Human U266 cells at 3.0 x 10^6^ cells/mL were cultured with Formononetin at a 10 µg/mL concentration for 72 hours. The cells were then harvested, and the supernatant was removed by centrifugation at 3000 rpm for 5 minutes. The cells’ suspension was washed with PBS at 3000 rpm for 5 minutes. Protein extraction was performed through cell lysis using RIPA lysis buffer, following the manufacturer’s instructions (Santa Cruz Biotechnology, Santa Cruz, CA). To extract the protein, 100-200 µl of RIPA lysis buffer was added and vigorously vortexed while incubating on ice for about 1 hour. The mixture was then centrifuged at 14,000 rpm for 20 minutes at 4°C. The isolated protein was used for standard western blot analysis. Gel electrophoresis was conducted at 90 V for 15 minutes, followed by 100–110 V for the remaining time. After running the gel, the proteins were transferred to a PVDF membrane. The membrane was incubated overnight at 4°C with primary antibodies of IκBα, NF-ĸB, and GAPDH. After overnight incubation with the primary antibodies, the membrane was washed with PBST the next day and then incubated with secondary antibodies for 2 hours. The primary antibodies of IκBα, NF-κB, and GAPDH were used at a 1:1,000 dilution and were ordered from Cell Signaling (Danvers, MA, USA). The secondary antibodies from Cell Signaling were used at a 1:10,000 dilution.

### 2.10 Statistics

We utilized GraphPad Prism software (version 9, GraphPad Software, Inc., San Diego, CA) to perform statistical analyses. To compare the differences between two groups, we conducted one-way ANOVA (analysis of variance) followed by Bonferroni correction. We considered a p-value of ≤ 0.05 as statistically significant.

## 3 Results

### 3.1 Formononetin decreased IgE production in plasma B cell line and peripheral blood mononuclear cells

We cultured formononetin at varying concentrations of 20, 10, 5, and 2.5 µg/mL. Our results showed that formononetin significantly reduced IgE in a dose-dependent manner in human myeloma cells compared to the control group (*p* < 0.001; **Figure 1A**). The percentage inhibition ranges for different concentrations are as follows: 2.5 µg/mL (44% - 56%), 5 µg/mL (56% - 62%), 10 µg/mL (70% - 76%), and 20 µg/mL (84% - 88%). Furthermore, cell viability data indicated that no toxic effects were observed across the tested concentrations (**Figure 1B**). Next, we determined whether formononetin can inhibit IgE production in human peripheral blood mononuclear cells (hPBMCs) taken from food-allergic patients. We cultured formononetin at 20 and 10 µg/mL concentrations with hPBMCs for 72 hours to test this. Our results show that formononetin significantly decreased IgE production from hPBMCs at concentrations of 20 and 10 µg/mL (***p < 0.001 vs control, **Figure 1C**). The percentage inhibition ranges for different concentrations are 2.5 µg/mL (70% - 80%) and 5 µg/mL (82.5% - 85%). Moreover, cell viability data indicate no toxicity across the tested concentrations (**Figure 1D**).

### 3.2 Formononetin decreased Beta-hexosaminidase release in basophil cell lines RBL-2H3 and KU812 cells

**Figure 2:**
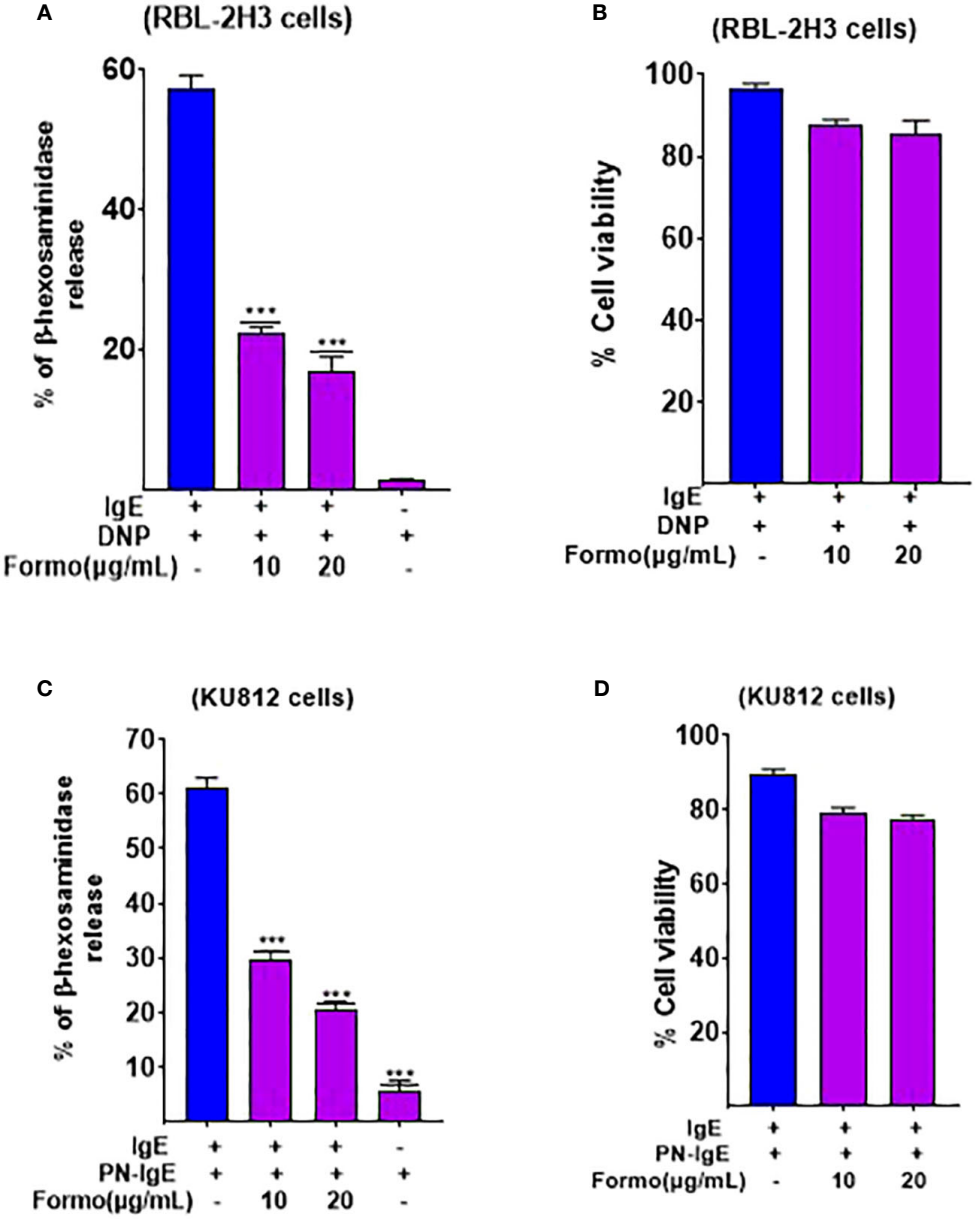
Formononetin decreases Beta Hexosaminidase release in basophil cell line RBL-2H3 and KU812 cells. Beta hexosaminidase release was measured in basophil cell lines **(A)**. RBL-2H3 after 24 hours incubation with formononetin at different concentrations of 10, 20 µg/mL **(B)**. Cell viability data of RBL-2H3 after 24 hours incubation with formononetin **(C)**. KU812 cells after 24 hours of incubation with formononetin 10, 20 µg/mL. **(D)** Cell viability data of KU812 cells after 24 hours incubation with formononetin. Data represents triplicate experiments and expressed as mean ±SD. *** p<0.001 vs control (0.1%DMSO). Formo, Formononetin.

The RBL-2H3 cell line, also known as rat basophilic leukemic cells, and KU812 human basophilic chronic myelogenous leukemia cells have been extensively used in allergic research. They are commonly utilized to study the effect of substances on mast cell degranulation, activation, and other mast cell-related biological functions. We used these cells to investigate the effect of formononetin on mast cell function, specifically degranulation. We cultured formononetin at 10 and 20 µg/mL concentrations with RBL-2H3 cells for 24 and 48 hours and with KU812 cells for 24 hours only. After incubation, we sensitized the cells with anti-DNP IgE for RBL-2H3 cells and serum collected from patients with peanut allergy. Our results indicated that formononetin at 10 and 20 µg/mL concentrations significantly reduced beta-hexosaminidase release (p < 0.001, **Figure 2A**) within 24 and 48 hours in RBL-2H3 cells with no cytotoxicity (**Figure 2B**). We observed a similar effect in KU812 cells, where formononetin at 10 and 20 µg/mL concentrations also significantly decreased beta-hexosaminidase release (p < 0.001, **Figure 2C**) with no cytotoxicity (**Figure 2D**).

### 3.3 Formononetin regulates therapeutic targets in food allergy, IgE and mast cell diseases

**Figure 3:**
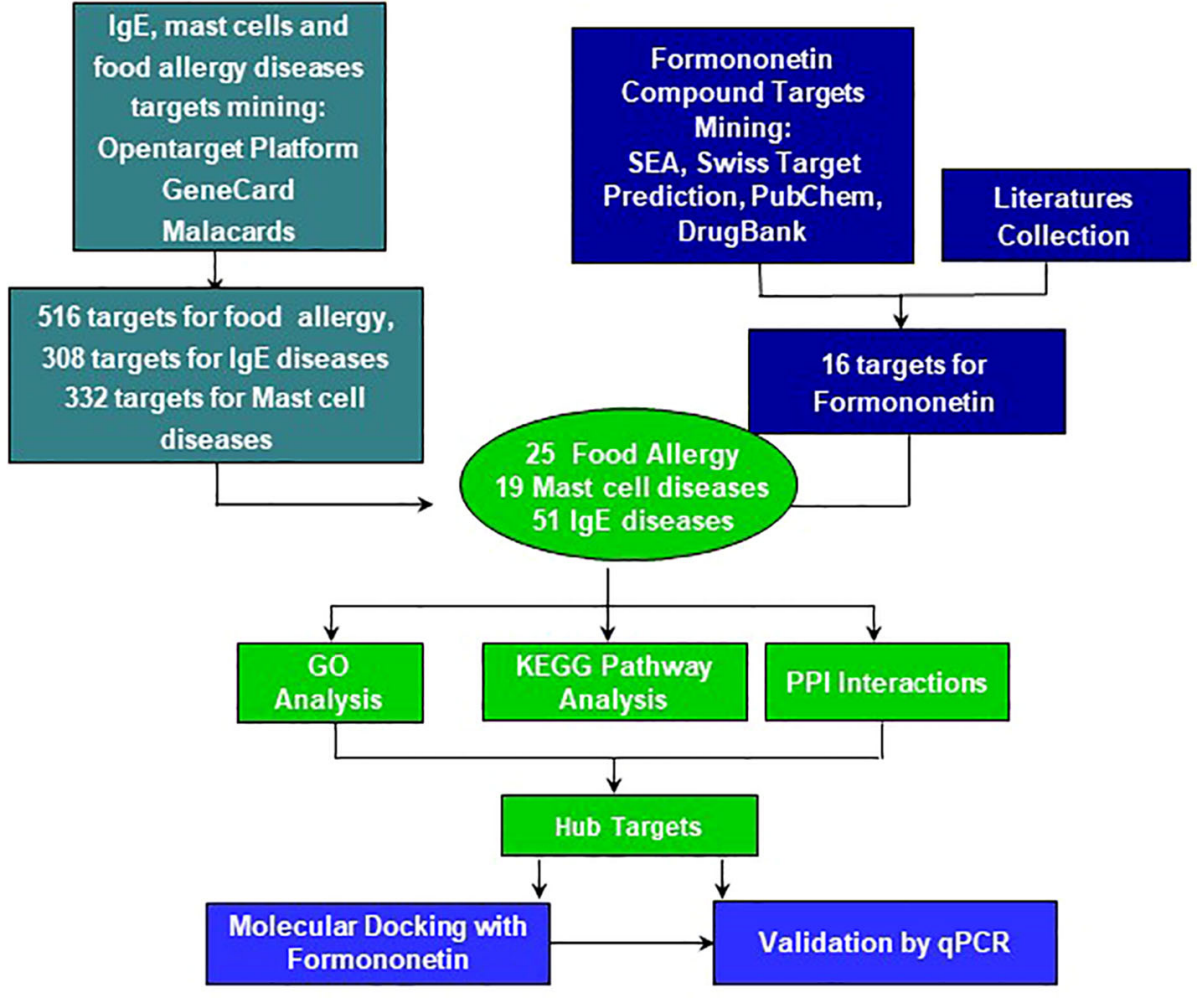
The workflow of computational modeling used for analysis of Formononetin for treatment of food allergy, mast cell diseases and IgE diseases. First, total 516 genes for Food allergy, 308 genes for IgE diseases and 332 genes for Mast cell diseases were selected as their targets. 109 inflammatory and biological targets of formononetin were collected based on literatures and following published databases: swiss target prediction, SEA, pub-chem and drug-bank. Mining formononetin targets into obtained disease targets uncovers that formononetin might potentially regulate 25 targets of food allergy, 51 target of IgE diseases and 19 targets of mast cell diseases. SEA, similarity ensemble approach; PPI, protein-protein Interactions, GO, gene ontology, KEGG, Kyoto encyclopedia of genes and genomes.

**Figure 4:**
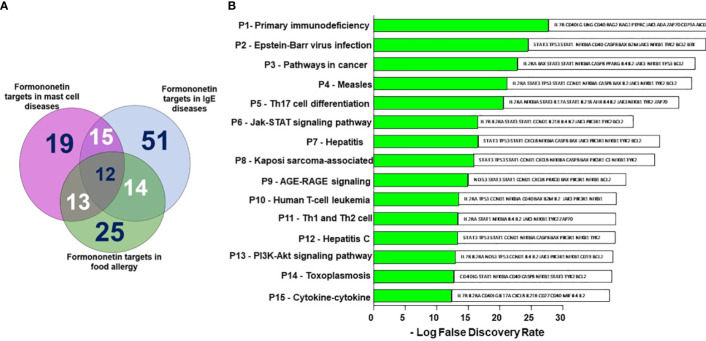
Shared formononetin targets and identification of targets by KEGG pathway analysis. **(A)** Venn diagram showing the number of targets predicted using computational modeling. Among them 12 targets were shared between mast cell diseases, IgE diseases and food allergy. **(B)** KEGG Pathway analysis. Y-axis: The top 15 pathway relevant to the enriched targets (left) and genes associated with each pathways (right); X-axis: the significance of each term ranked with-Log FDR. P, Pathway; FDR, False Discovery Rate.

We obtained 516 therapeutic targets for food allergy, 308 for IgE, and 332 for mast cell diseases from disease biological target databases such as Opentarget platform, GeneCard and Malacard (**Figure 3**). Additionally, from compound biological target databases such as SEA, Swiss target prediction, PubChem, and literature, we found 106 biological targets of formononetin. We then analyzed these biological targets of formononetin to identify the targets specific to food allergy, IgE diseases, and mast cell diseases. This resulted in 25 targets for food allergy, 51 for IgE diseases, and 19 for mast cell diseases. (**Figure 4A**). We further analyzed these targets using gene ontology, KEGG pathway analysis, and CTPD network and protein-protein interaction.

### 3.4 KEGG pathway identified top pathways relevant in food anaphylaxis regulated by formononetin

We have identified the top 15 pathways relevant to the enriched targets based on **Figure 4B**. The primary immunodeficiency pathway appears to be the most enriched pathway among the targets, although it is unrelated to food allergy. People with primary immunodeficiency disorders typically have a weakened immune system, increasing their risk of food allergy. On the other hand, pathways such as Epstein-Barr virus infection, Measles pathway, T cell differentiated pathways Th1, Th2, and Th17, Jak-STAT, AGE-RAGE, and PI3K-Akt pathways are all linked to food allergy (**Figure 4B**).

### 3.5 Gene ontology shows biological processes and functions regulated by formononetin

The top 15 enriched gene ontology analyses were selected based on the biological process and GO terms with a False Discovery Rate (FDR) < 0.01 were ranked by enrichment score (-Log FDR) in (**Supplementary Figure 3**). The biological processes regulated by formononetin are related to B cell development, differentiation, and activation. B cell activation occurs when specific cytokines are delivered from activated T cells and when CD40L binds to the CD40 receptor on B cells (45–48). Activation of the CD40 receptor by CD40L is sufficient to drive B cell proliferation (49), but the presence of soluble cytokines increases proliferation and initiates B-class switch recombination in B cells (50). Cytokines such as IL-2, IL-4, and IL-10 aid B cell proliferation and differentiation (51). CD27 was found to be a formononetin target that appeared in multiple GO pathways. Therefore, understanding the interaction between formononetin and CD27 is important and would be a good area for future studies.

### 3.6 Compound-target-pathway-disease network of targets regulated by formononetin

**Figure 5:**
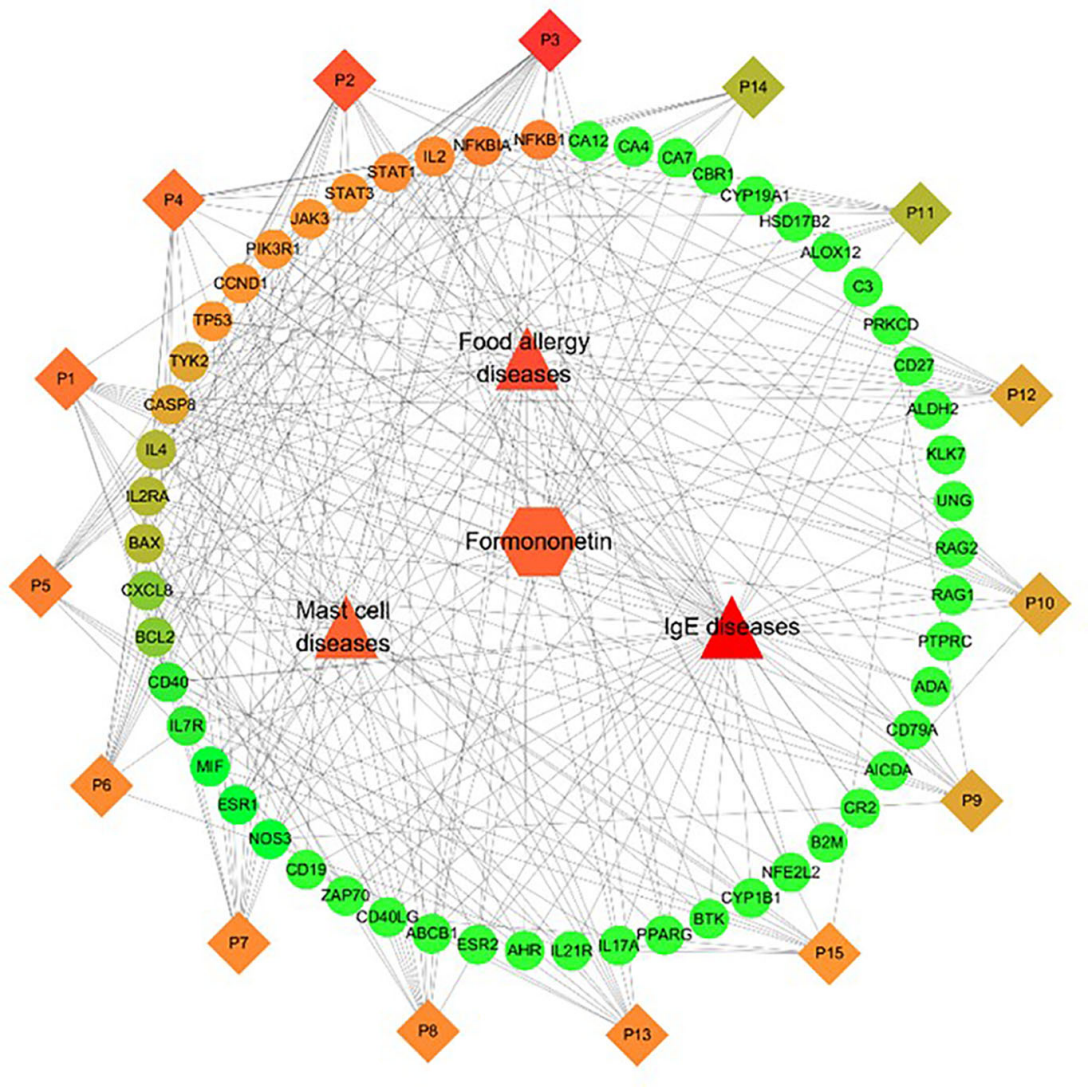
Identification of Compound-Target-Pathway-Disease (C-T-P-D) network of the formononetin in food allergy, IgE and mast cell diseases. Diamonds, circles, squares, triangles represent formononetin, common targets, pathways and diseases, respectively. Node size and node color from green to red is proportional to its degree. Black lines stands for interaction between nodes.

The C-T-P-D network which contains formononetin, targets, and top 15 pathways in food allergy, IgE, and mast cell diseases provides information about the potential pharmacological mechanisms of formononetin for preventing and treating food anaphylaxis at the molecular level (as shown in **Figure 5**). The frequency of targets appearing in the top 15 pathways implies their influence and importance. Nodes with colors ranging from orange to green are proportional to the degree value, displaying their importance from high to low in the network. Targets such as NFKB1, NFKBIA, IL2, STAT1, STAT3, JAK3, PIK3R1, CCND1, TP53, TYK2, and CASP8 have a high degree of importance, IL4, IL2RA, and BAX have a medium degree of importance, while the other targets in green have low degrees of importance. These targets are mapped to the P6 - Jak-STAT signaling pathway and the P13 - PI3K-Akt signaling pathway.

### 3.7 Protein targets regulated by formononetin identified by protein-protein network

**Figure 6:**
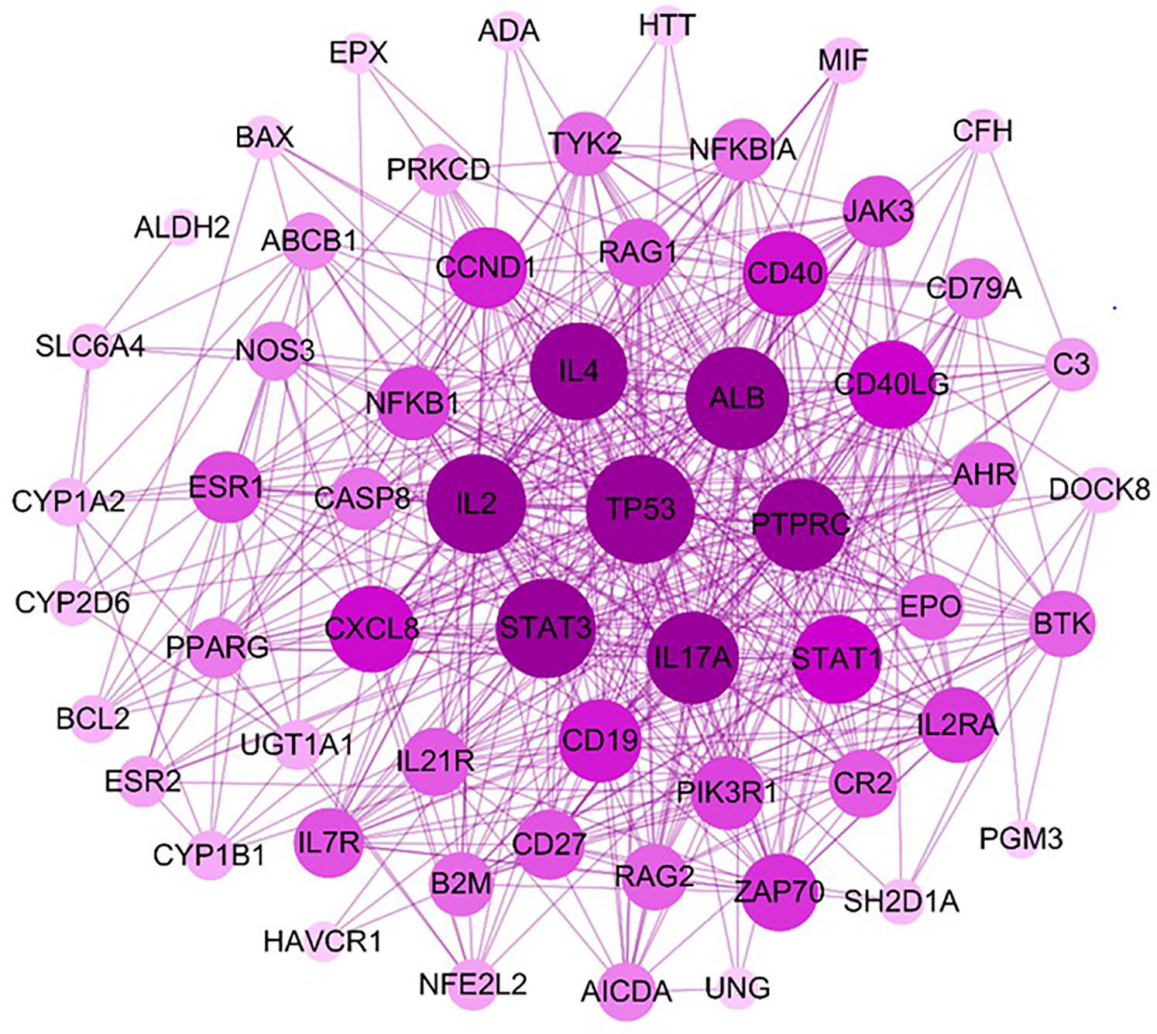
Identification of high frequent interactions among the targeted proteins by PPI network. The PPI network was constructed by mapping potential targets to the Strings database. The size of the node from large to small is proportional to its degree value in the network. The circles represent the therapeutic targets, and the purple lines represent the interaction between the nodes. PPI, Protein- Protein Interaction.

Protein-protein networks play a significant role in defining the molecular basis of diseases. **Figure 6** illustrates the network of target proteins of formononetin in food allergy, IgE, and mast cell diseases. The size of each node corresponds to its degree value in the network. The circles in the figure represent the therapeutic targets of formononetin for the mentioned diseases, while the purple lines represent the interaction between the therapeutic targets. The therapeutic targets with the largest node size, such as TP53, ALB, IL2, IL4, STAT3, PTPRC, and IL17A, are identified as the therapeutic targets with the highest degree of interaction (**Figure 6**). Other relevant therapeutic targets with medium-sized nodes include IL17A, CXCL8, CASP8, NFKB1, CCND1, RAG1, CD40, CD40LG, AHR, EPO, BTK, and IL2RA. Studies have shown that increased levels of TP53 mRNA occur during B cell differentiation, indicating its significance as a key target for B cell differentiation (52) TP53 has also been found to negatively regulate IgE-mediated mast cell activation through interaction with the NF-κB pathways (53). TP53 is upregulated in mast cells activated by IgE crosslinking, while TP53-deficient mast cells were shown to have increased IgE-mediated degranulation (54).

### 3.8 Formononetin regulates the gene expression of molecular targets of IgE regulation in IgE producing human myeloma cells

**Figure 7:**
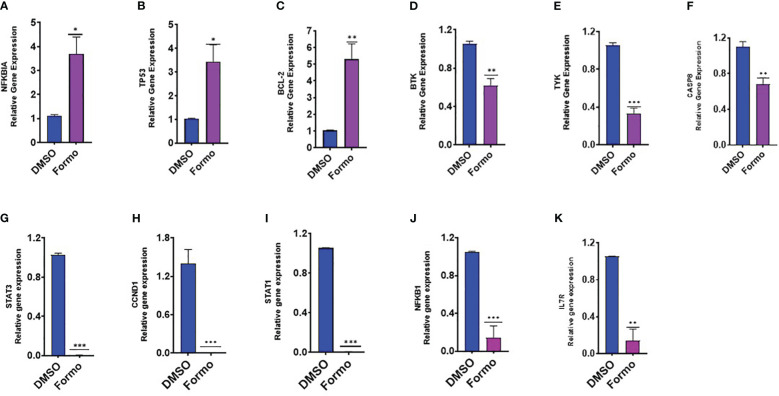
Effect of Formononetin on the gene expression of select molecular targets on IgE regulation. IgE-producing human myeloma cells were cultured with Formononetin for 72 hours. mRNA expression was determined using qRT-PCR. **(A)** NFKBIA **(B)** TP53 **(C)** BCL-2 **(D)** BTK **(E)** TYK **(F)** CASP8 **(G)** STAT3 **(H)** CCND1 **(I)** STATI **(J)** NFKB1 **(K)** IL7RA. Data represents triplicate experiments and expressed as mean ±SD. *p<0.05; **p<0.01, ***p<0.001 vs control (0.1%DMSO). *Formo, Formononetin.

Formononetin has been found to inhibit IgE production in both IgE producing human myeloma cells and peripheral blood mononuclear cells collected from patients with food allergies. Using computational methodology, we have identified targets in IgE diseases, mast cell diseases and food allergy that are highly regulated by formononetin. To determine the effect of formononetin on these targets, we cultured cells with a concentration of 10 µg/mL for 72 hours, followed by measuring the gene expression of selected targets with quantitative polymerase chain reaction qRT-PCR. After isolating RNA from the harvested cells using the RNesy QIAGEN kit, we quantified the RNA using the NanoDrop™ UV-Vis Spectrophotometer. Formononetin was found to significantly increase the gene expression of NFKBIA (p< 0.05), TP53 (p< 0.05), and BCL-2 (p< 0.01), while decreasing the gene expression of BTK (p< 0.01), TYK (p< 0.001), CASP8 (p< 0.01), STAT3 (p< 0.001), CCND1 (p< 0.001), STAT1 (p< 0.001), NFKB1 (p< 0.001), IL7R (p< 0.01) (**Figure 7**).

### 3.9 Formononetin regulates the gene expression of molecular targets in human basophil regulation

**Figure 8:**
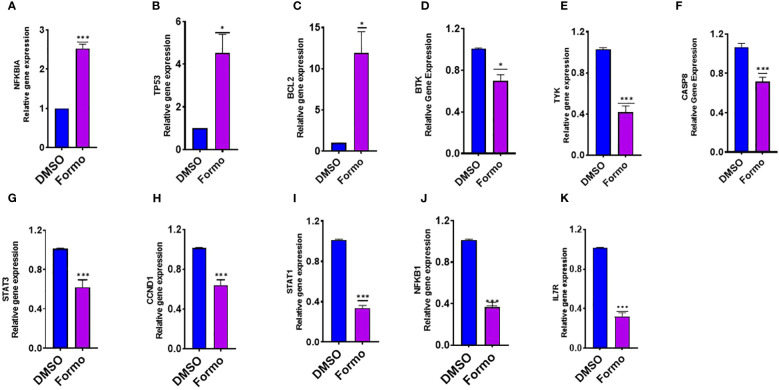
Effect of Formononetin on the gene expression of select molecular targets involved in regulating human basophil cells. Human Basophil cells, KU812, were cultured with Formononetin for 24 hours. mRNA expression was determined using qRT-PCR. **(A)** NFKBIA **(B)** TP53 **(C)**. BCL-2 **(D)** BTK **(E)** TYK **(F)** CASP8 **(G)** STAT3. **(H)** CCND1 **(I)** STAT1 **(J)** NFKB1 **(K)** IL7R. Data represents triplicate experiments and expressed as mean ±SD. *p<0.05; ***p<0.001 vs control (0.1%DMSO). Formo Formononetin.

RBL-2H3 and KU812 cells are commonly used as models for mast cells models for mast cell degranulation studies. In these studies, the beta-hexosaminidase release is used to measure mast cell degranulation. Formononetin has been shown to significantly reduce beta-hexosaminidase release in both RBL-2H3 (p< 0.001) and KU812 (p< 0.001) cells within 24 and 48 hours of culture. Through computational methods, we identified several targets highly regulated by formononetin in mast cell diseases, IgE diseases, and food allergies. To determine the effect of formononetin on these targets, we cultured KU812 cells with formononetin at a concentration of 10 µg/mL for 24 hours at a 1.0 × 10^6^ cells/mL. After the culture period, we harvested the KU812 cells and isolated RNA using the RNesy QIAGEN kit, then quantified the RNA using the NanoDrop™ UV-Vis Spectrophotometer. We then measured the relative gene expression of selected targets, including NFKBIA, TP53, BCL-2, BTK, TYK, and CASP8. Our results showed that formononetin significantly increased the gene expression of NFKBIA (p< 0.001), TP53 (p< 0.05), and BCL-2 (p< 0.05) while decreasing the gene expression of BTK (p< 0.05), TYK (p< 0.001), CASP8 (p< 0.001), STAT3 (p< 0.001), CCND1 (p< 0.001), STAT1 (p< 0.001), NFKB1 (p< 0.001), IL7R (p< 0.001) (**Figure 8**).

### 3.10 Formononetin regulates the protein expression of select molecular targets of IgE regulation in IgE-producing human myeloma cells

**Figure 9:**
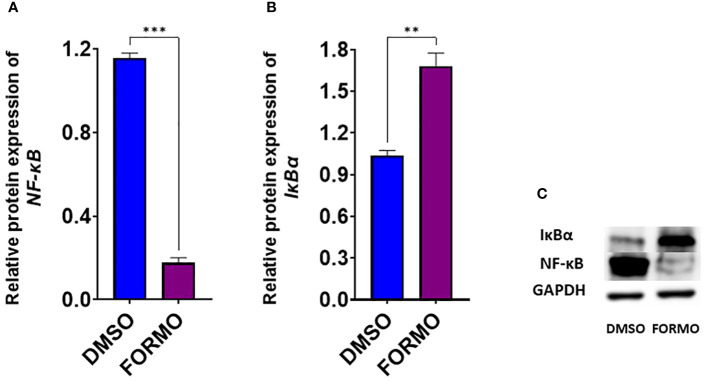
Effect of Formononetin on the protein expression of select molecular targets on IgE regulation. IgE-producing human myeloma cells were cultured with Formononetin at 10 µg/mL for 72 hours. Protein expression was determined using western blotting. Western blotting assay was conducted as described in the methods. Protein expression of **(A)**. NF-kB **(B)**. р-Іквα **(C)**. Western Bands. Data represents triplicate experiments and expressed as mean ±SD. **p<0.01; *** p<0.001 vs control (0.1%DMSO).

We found that formononetin regulates the gene expression of molecular targets in the PI3K/Akt and JAK/STAT pathways. Therefore, we determined the effect of formononetin on the protein levels of selected targets to see if it potentially regulates the targets at the protein level. Formononetin was found to regulate the gene expression of NFKB1 and NFKBIA. In particular, the gene expression of NFKB1 decreased while NFKBIA increased. NF-κB is important in the JAK/STAT and PI3/Akt pathways. We measured the protein levels of NF-κB and IκBα in U266 cells after treating them with formononetin at a concentration of 10 µg/mL for 72 hours. The results indicate that formononetin significantly increased the protein expression of IκBα (p<0.01) and decreased the protein expression of NF-κB (p<0.001) (**Figures 9A, B**).

## 4 Discussion and conclusion

Previous studies have shown that formononetin has the potential to be effective in treating allergic diseases by inhibiting mast cell degranulation and suppressing the IgE production from plasma B cells. Zi-Wen et al. (55) demonstrated that formononetin can inhibit mast cell degranulation in mice, thereby decreasing allergic responses by inhibiting IgE-independent mast cell degranulation and NF-KB signaling (55). Similarly, Yi et al. (56) showed the potential therapeutic effects of formononetin in an asthmatic mice model. Their study showed that formononetin reduced IL4, IL5, IL13, and IgE levels (56). Additionally, Yang et al. demonstrated that formononetin is the active compound in an anti-asthma herbal intervention formula (57). The anti-asthma herbal intervention formula has been shown to suppress IgE production in a murine model of asthma; Yang’s study identified formononetin as the major active compound responsible for this IgE suppressive effect.

In the study conducted by Yang et al. (3), it was reported that Formononetin, isolated from Sophora flavescentis, which is a component of an anti-asthma herbal intervention formula previously shown to be a potential treatment for allergic asthma in a murine model, exhibited an IgE suppressive effect plasma B cell line. This effect was observed over a 2.0 -20 µg/mL concentration range without any signs of cytotoxicity. Furthermore, previous studies by Yang et al. (11) also demonstrated that Berberine and Limonin had a high safety profile when tested at concentrations up to 10 µg/mL in peripheral blood mononuclear cells, showing no toxicity. Zi-Wen et al. (55) demonstrated that formononetin at concentrations up to 100 µM displayed no signs of toxicity in RBL-2H3 cells. Consistent with the findings from previous studies, our study tested formononetin over a concentration range of 2.5 to 20 ug/mL in plasma U266 cells, PBMCs, and mast cell lines RBL-2H3, KU812 cells, we similarly observed no significant cytotoxicity.

This study showed that formononetin can potentially reduce Immunoglobulin E levels in human PBMCs. Additionally, we found that the compound can decrease the degranulation of mast cells. We showed that formononetin significantly decreased beta-hexosaminidase release in both RBL-2H3 cells and KU812 cells. We are the first to report these two effects of formononetin in human PBMCs, RBL-2H3 cells and KU812 cells. To determine the mechanism of formononetin regulation, we employed computational technology. Our study revealed that formononetin has the potential to regulate 25 targets in food allergy, 51 in IgE diseases, and 19 in mast cell diseases. We analyzed these targets in the KEGG pathway and selected the top 15 pathways. Among the top 15 pathways, we focused on the JAK-STAT signaling pathways because the targets mapped to these pathways are highly linked with the development of food anaphylaxis. Primary immunodeficiency, Epstein-Barr virus infection, measles virus infection, and hepatitis B and C virus infection are also included in the top 15 pathways. We used gene ontology to identify the potential biological mechanisms formononetin regulates in B cells and Mast cell diseases. Our findings show that formononetin regulates genes involved in receptor signaling, activation, proliferation, and class switch recombination in B cells. In Mast cells, formononetin targets mast cell activation and degranulation genes. The genes ADA, BTK, PI3K, and PRKCD appear in multiple GO terms, indicating their importance in mast cell activation and degranulation. However, further *in vitro* and *in vivo* studies are necessary to understand how formononetin interacts with these genes. We also found that formononetin regulates CD40L and cytokines that activate B cells, initiate class switching, and augment IgE secretion. Cytokines such as IL-2, IL-4, and IL-10 promote B cell proliferation and differentiation (51)IL-4 and CD-40 cross-linking play crucial roles in IgE secretion, while IL-2 and IL-10 induce IgM, IgG, and IgA secretion (58–60).

The pathological effects of IgE are mainly caused by plasma IgE+ B cells, which have a longer lifespan of months to years compared to memory IgE B cells, which typically live for about 10-12 days. The differentiation of B cells involves critical pathways such as the PI3K and NF-kB pathways, which are regulated by formononetin. This study demonstrates that formononetin regulates targets in the PI3K pathway, indicating its potential role in regulating B cell differentiation. However, further research is needed to determine the antiproliferative effects of formononetin on plasma and memory IgE B cells.

Furthermore, we validated the computational results, and our data showed that formononetin significantly decreased the expression of IL7R, NFKB1, STAT1, CCND1, STAT3, CASP8, TYK, and BTK, while it significantly increased the expression of BCL2, NFKBIA, P53 In both plasma B cell line U266 cells and human basophil cell line KU812 cells. Suzuki et al. showed that tumor suppressor functions as a negative regulator in IgE-mediated mast cell activation (53). The results of molecular docking analysis on TYK, BTK, P53, BCL-2, NFKBIA, and CASP8 targets revealed that formononetin has a strong affinity and interaction with these targets, as demonstrated in **Supplementary Figure 2** and **Supplementary Table 2**. This data indicates that formononetin directly binds to these proteins and regulates their functions, thus affecting the biological processes of B cells and mast cells.

A study by Yang et al. found that formononetin could be a potential therapy for allergic asthma and other IgE-mediated diseases (57). The study suggested that formononetin inhibits B cell IgE production by regulating the ER stress transcription factor XBP-1. L. Zhang et al. demonstrated that formononetin might be beneficial in treating asthma by ameliorating airway inflammation in house dust mice (HDM)-challenged mice (61). They also revealed that Formononetin inhibits IgE production in HDM-challenged mice compared to the control group. Similarly, we have shown that formononetin decreased IgE production in plasma B cell line U266 cells and human peripheral blood mononuclear cells collected from patients with food allergies. This further demonstrates the potential for formononetin to treat IgE-mediated diseases such as IgE-mediated asthma, atopic dermatitis, food anaphylaxis, and allergy, requiring further investigation.

The options for treating food allergies are limited, and researchers continuously seek better treatment methods. Non-allergen-specific immunotherapies such as omalizumab (anti-IgE), dupilumab (anti-IL4), and ekotimab (anti-IL-33) have been utilized in the treatment of allergic diseases and food allergies (62, 63). However, there is limited evidence to show that they can offer long-term benefits in altering food allergy process. Therapeutics capable of inhibiting IgE production from plasma B cells have the potential to provide long-term protection against food anaphylaxis. Our study, in conjunction with previous research conducted by Yang et al. (57) shows that formononetin can decrease the production of IgE in plasma B cells. Furthermore, this study demonstrated potential mechanisms, pharmacological targets, pathways, and regulated genes. As a result, formononetin could be a potential long-term protection treatment.

These findings of formononetin provide a comprehensive overview of how it can be a potential treatment candidate for food anaphylaxis in food allergy. By inhibiting the activation, proliferation, and differentiation of B cells and inhibiting mast cell activation and degranulation, formononetin can be used as a potential treatment and preventive option for food anaphylaxis.

## Data Availability

The datasets presented in this study can be found in online repositories. The names of the repository/repositories and accession number(s) can be found in the article/**Supplementary Material**.
