# Supplementary figures and images for: Formononetin inhibits IgE by huPlasma/PBMCs and mast cells/basophil activation via JAK/STAT/PI3-Akt pathways
Source: Front Immunol. 2024 Aug 15;15:1427563. doi: 10.3389/fimmu.2024.1427563 (PMC11363073; doi:10.3389/fimmu.2024.1427563)

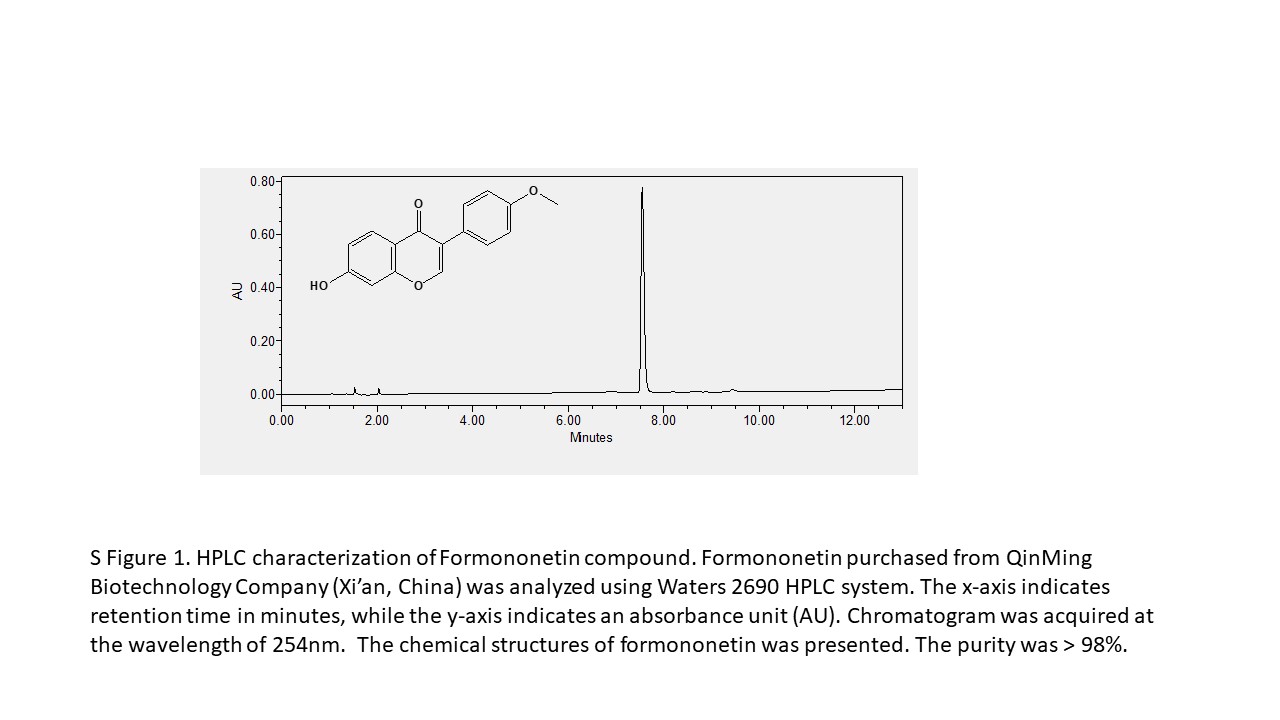

Supplement: Supplementary file 1 [file Image1.jpeg]

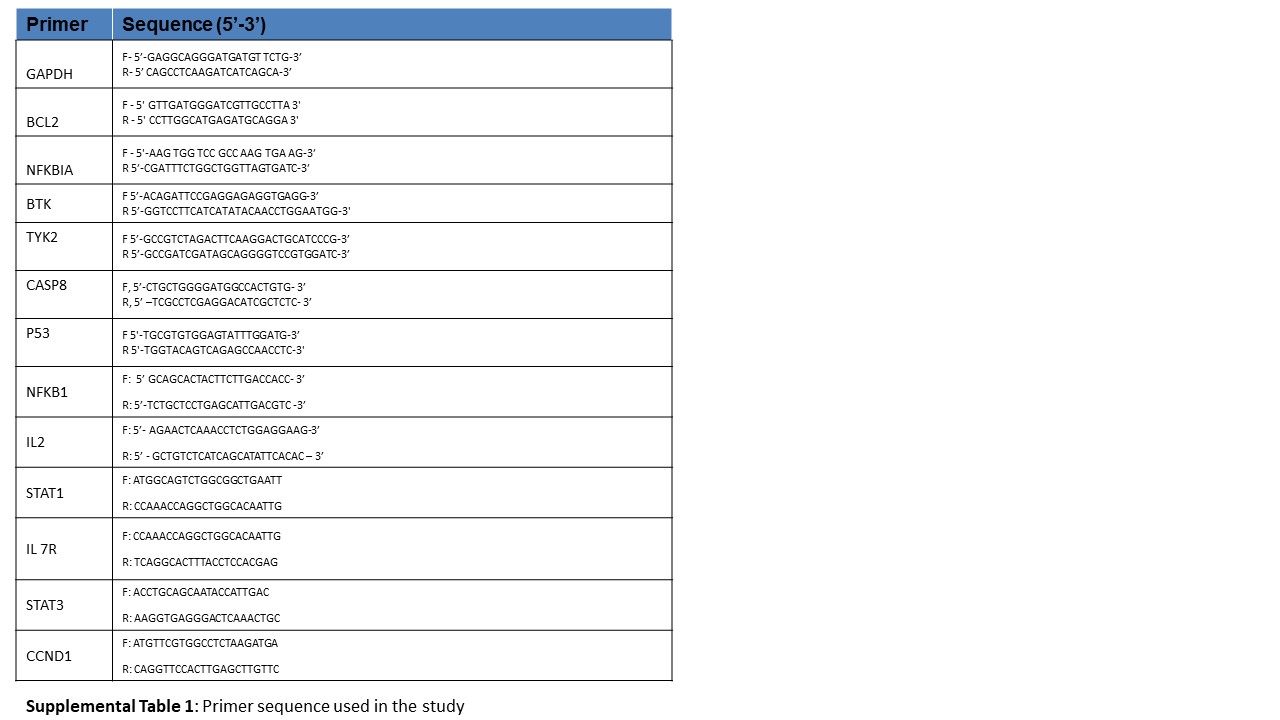

Supplement: Supplementary file 2 [file Image2.jpeg]

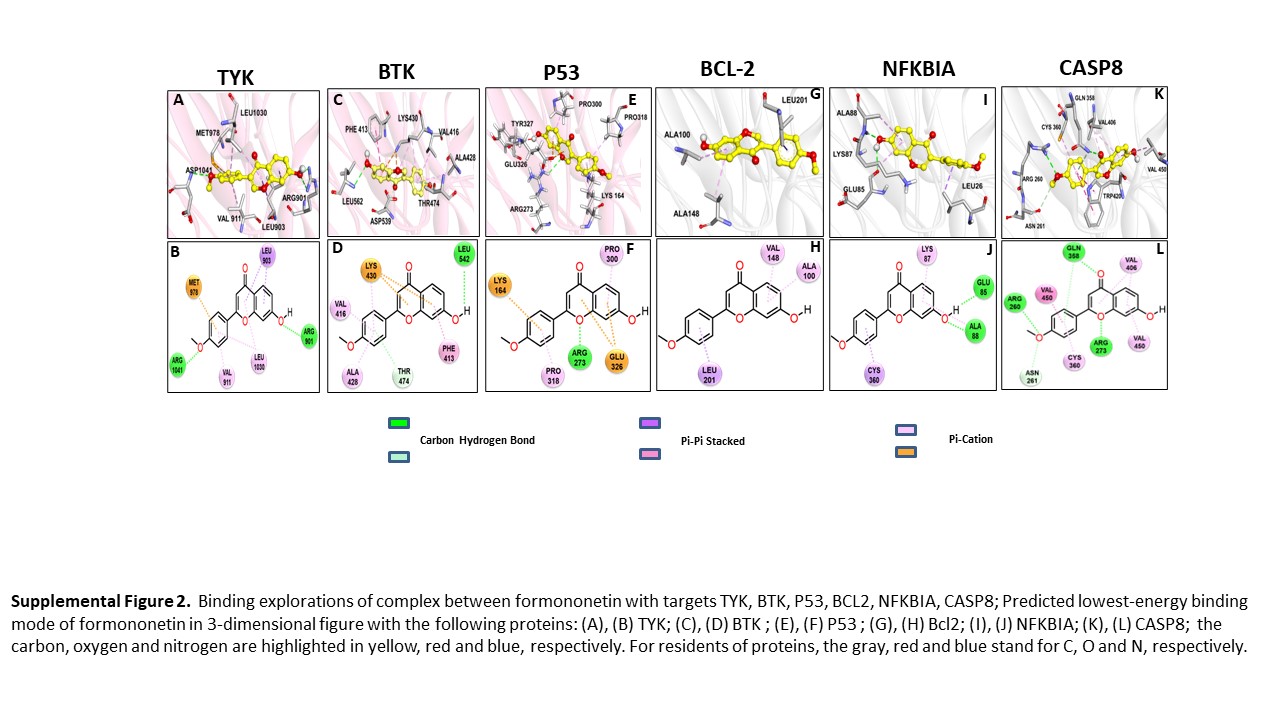

Supplement: Supplementary file 3 [file Image3.jpeg]

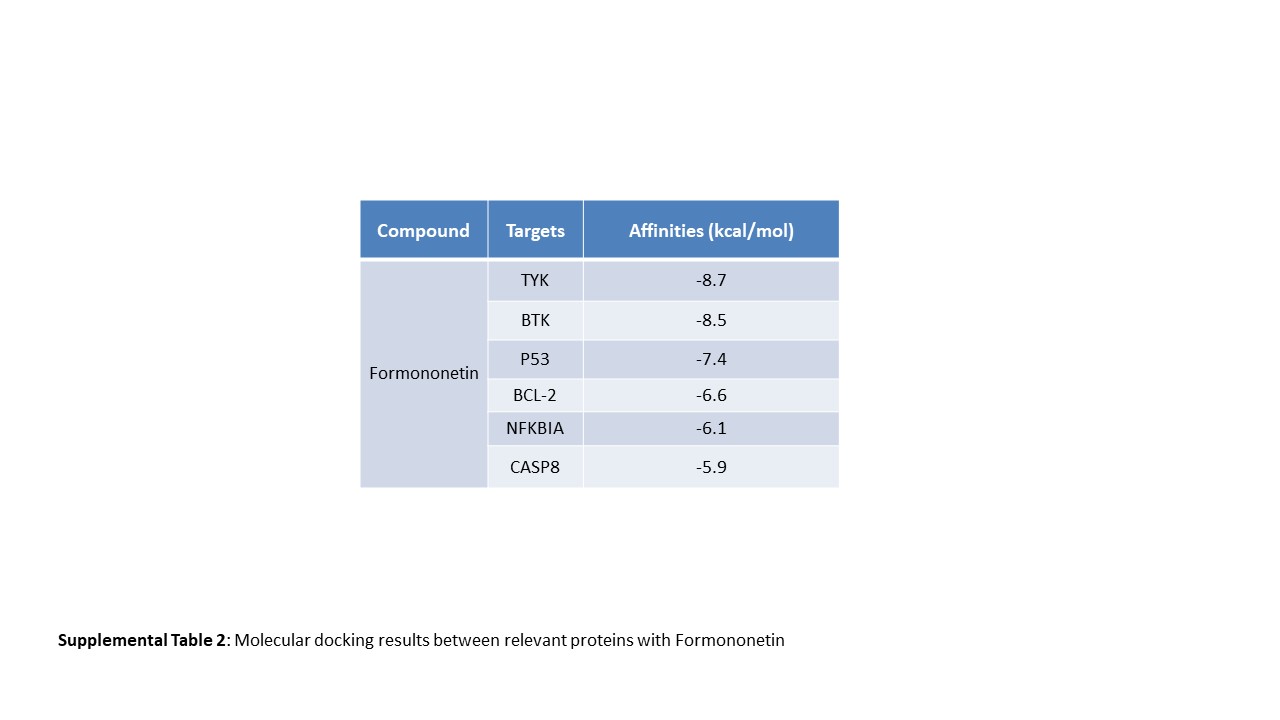

Supplement: Supplementary file 4 [file Image4.jpeg]

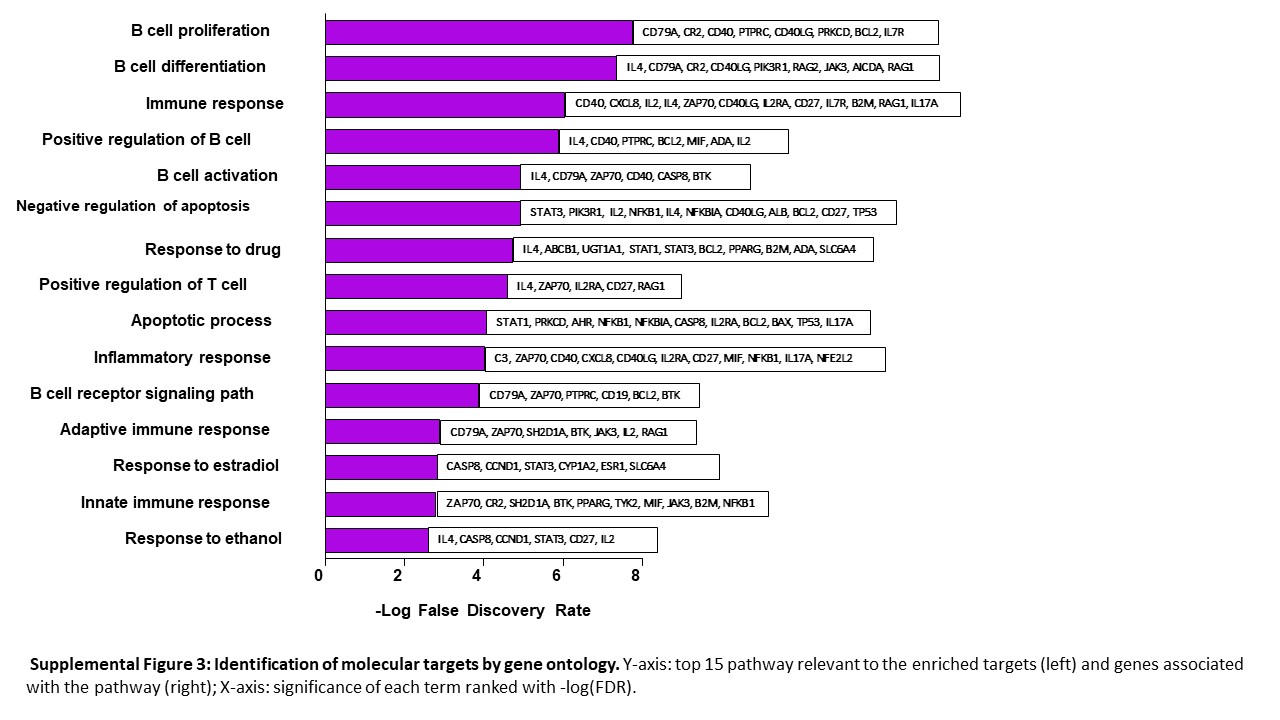

Supplement: Supplementary file 5 [file Image5.jpeg]
